# Radiobiological characterisation of a 28 MeV proton beam delivered by the MC-40 cyclotron

**DOI:** 10.1038/s41420-025-02635-1

**Published:** 2025-07-21

**Authors:** Maria Rita Fabbrizi, Jonathan R. Hughes, Leah D. Punshon, Laura Hawkins, Vasily Sorokin, Alice Ormrod, Emma Melia, Karthik Vaidya, Carlos P. Rubbi, Ben Phoenix, Mark A. Hill, Jason L. Parsons

**Affiliations:** 1 Department of Cancer and Genomic Sciences, University of Birmingham, Edgbaston, Birmingham, UK; 2 Oxford Institute for Radiation Oncology, University of Oxford, Gray Laboratories, ORCRB Roosevelt Drive, Oxford, UK; 3 Medical School, Edge Hill University, St Helens Road, Ormskirk, UK; 4 School of Physics and Astronomy, University of Birmingham, Edgbaston, Birmingham, UK

**Keywords:** Cell death, Cancer therapy

## Abstract

Proton beam therapy (PBT) is a targeted radiotherapy treatment that can deliver the majority of the radiation dose to the tumour being treated via the Bragg peak. However, there is biological and clinical uncertainty of PBT due to the increases in linear energy transfer (LET) at and around the Bragg peak. Through radiobiological characterisation of a 28 MeV pristine proton beam at several positions relative to the Bragg peak, we demonstrate that there are decreases in survival of head and neck squamous cell carcinoma (HNSCC) and HeLa cells relative to increasing LET. Through monitoring DNA damage using γH2AX/53BP1/OGG1 foci via immunofluorescence microscopy and different versions of the comet assay, we show that increasing relative biological effectiveness (RBE) is directly associated with predominantly DNA single strand breaks that were more difficult to repair and persisted, in addition to a strong correlation with increases in the presence of more persistent complex DNA damage. Increasing frequencies of micronuclei as a marker of chromosomal damage were also observed as a function of LET. Our data demonstrate that increases in LET across the Bragg peak can create changes in the DNA damage spectrum that drive the radiobiological response.

## Introduction

Proton beam therapy (PBT) is a precision-targeted radiotherapy treatment that can generate optimal tumour targeting whilst minimising the radiation dose to the associated normal tissues and organs at risk proximal to the tumour [1]. This is achieved through a greater depth-dose deposition profile in comparison to conventional X-ray irradiation, where a low entrance dose is followed by maximal energy deposition in a finite region known as the Bragg peak, ensued by a low exit dose. However, the Bragg peak is associated with increases in linear energy transfer (LET) at and around the Bragg peak, and particularly towards the distal end. This variable LET across the radiation track leads to biological and clinical uncertainty, although a relative biological effectiveness (RBE) of 1.1 is still used in treatment planning, which has been highly debated [2, 3]. This is due to the fact that a major consequence of the increased LET is the clustering of ionisation events [4, 5], which can have a different impact on critical biomolecules, particularly DNA, compared to low-LET radiation. Indeed, the formation of complex DNA damage (CDD), defined as two or more DNA lesions within 1–2 helical turns of the DNA, is considered a major contributor to the enhanced RBE of PBT, and of the cell killing effects of ionising radiation in general [6]. Despite this, we surprisingly still have little information characterising the radiobiological responses of proton beams relative to LET on appropriate cellular model systems.

In general, it is considered that low-LET (or high energy) PBT generates a spectrum of DNA damage that is similar to X-ray irradiation, whereas high-LET (or relatively low energy) PBT will specifically lead to increased levels and persistence of CDD that causes cell lethality. This has been predicted through mathematical modelling [7–9], where for example it has been shown that low-LET radiation can generate ~30% CDD but this increases to ~90% for very high-LET α-particles. However, analysing CDD in biological systems has proved challenging to measure and quantitate making it difficult to specifically analyse the cellular response to PBT at different energies/LET [6, 10]. Previous studies have shown this indirectly by demonstrating that increased LET across the proton Bragg peak leads to a reduced cell survival and therefore a higher RBE [11–13]. Furthermore, and using 53BP1 foci as a marker of DNA double strand breaks (DSBs), it has been shown that there are persistent foci at the distal end of a spread-out Bragg peak, indicative of CDD formation [14]. A robust and systematic analysis of RBE as a function of depth across the proton Bragg peak and the impact on DNA damage and the levels of complexity has therefore not been completed.

Using the MC-40 cyclotron, we have analysed the RBE of a 28 MeV pristine beam comparing the entrance dose with different positions across the Bragg peak in HeLa and two head and neck squamous cell carcinoma (HNSCC) cell lines. We correlated RBE values with the levels and repair of DNA single and double strand breaks (SSB and DSB), and importantly to CDD, but also to chromosomal damage (micronuclei). We find that there are significant increases in the levels of persistent CDD and micronuclei generated at the Bragg peak that are a driving factor towards the enhanced RBE of PBT.

## Results

### Increased RBE as a function of LET across a pristine proton Bragg peak

**Fig. 1:**
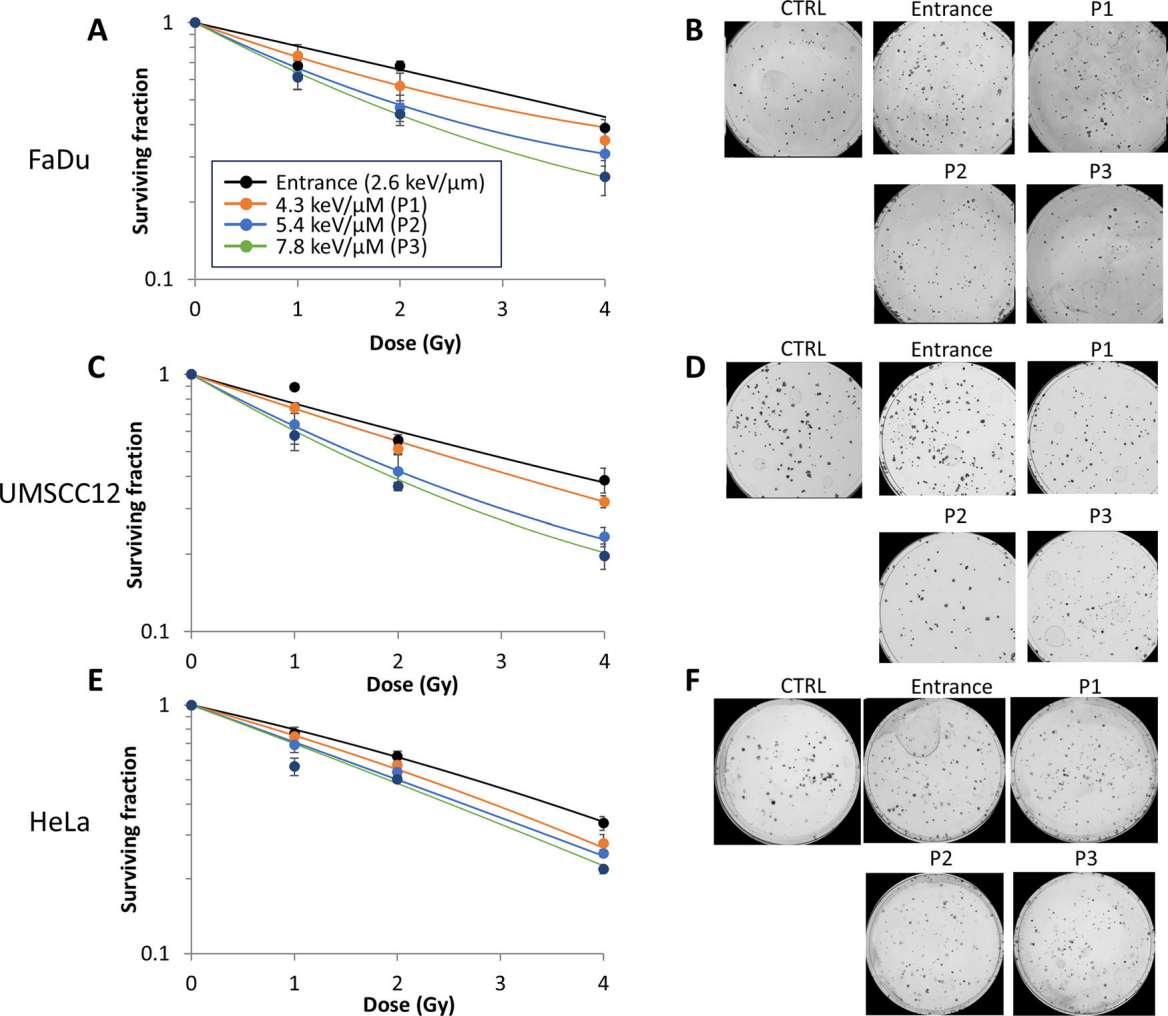
Protons with increasing LET lead to enhanced RBE in HeLa and HNSCC cells. **A** and **B** FaDu, **C** and **D** UMSCC12, and **E** and **F** HeLa cells were irradiated with entrance dose 28 MeV protons or the different positions across a pristine Bragg peak with increasing LET. Clonogenic survival of cells was then analysed from three biologically independent experiments. (**A**, **C** and **E**) Shown is the mean surviving fraction±S.E. (**B**, **D** and **F**) Representative images of colonies formed from unirradiated (Control) cells, and those following a 2 Gy PBT dose (double the numbers of cells seeded).

We first analysed the impact of a 28 MeV pristine proton beam delivered at different positions (and therefore LETs) relative to the Bragg peak on the survival of HeLa and two HNSCC cell lines (UMSCC12 and FaDu). Compared to entrance dose protons (2.6 keV/µm), we examined three positions relative to the Bragg peak (corresponding to LETs of 4.3, 5.4 and 7.8 keV/µm, respectively; Supplementary Fig. S1A–I). We observed an LET-dependent reduction in clonogenic survival of all cell lines tested (Fig. 1A–F and Supplementary Fig. S2A-C). RBE values calculated at 50% survival (Table 1) increased from 1.19–1.27 (4.3 keV/µm), 1.37–1.79 (5.4 keV/µm) and 1.44–2.02 (7.8 keV/µm) compared to entrance dose values (2.6 keV/µm). It should be noted that using linear quadratic (LQ) analysis, the two positions delivering the highest relative LET (5.4 and 7.8 keV/µm) gave surviving fractions that were significantly different from the entrance dose in all three cell lines (Table 2). Interestingly, HeLa cells were notably less responsive to LET-dependent effects than the HNSCC cell lines, as shown through lower RBE values. Nevertheless, these data demonstrate that the highest proton LET at the Bragg peak causes the greatest biological response in terms of cell killing.

**Table 1:** RBE values comparing protons of increasing LET versus entrance dose protons in HeLa and UMSCC cell lines.

		Cell line	
Treatment	FaDu	UMSCC12	HeLa
P1	1.27	1.19	1.20
P2	1.78	1.79	1.37
P3	2.02	1.97	1.44

**Table 2:** Statistical analysis of clonogenic survival data comparing protons of increasing LET versus entrance dose protons in HeLa and UMSCC cell lines.

		Cell line	
Treatment	FaDu	UMSCC12	HeLa
P1	0.75	0.30	0.29
P2	0.006	0.001	0.05
P3	0.00015	0.000044	0.0046

Statistical analysis performed using linear quadratic (LQ) analysis.

### Increased proton LET leads to delays in the repair of DNA SSBs, but not DSBs

**Fig. 2:**
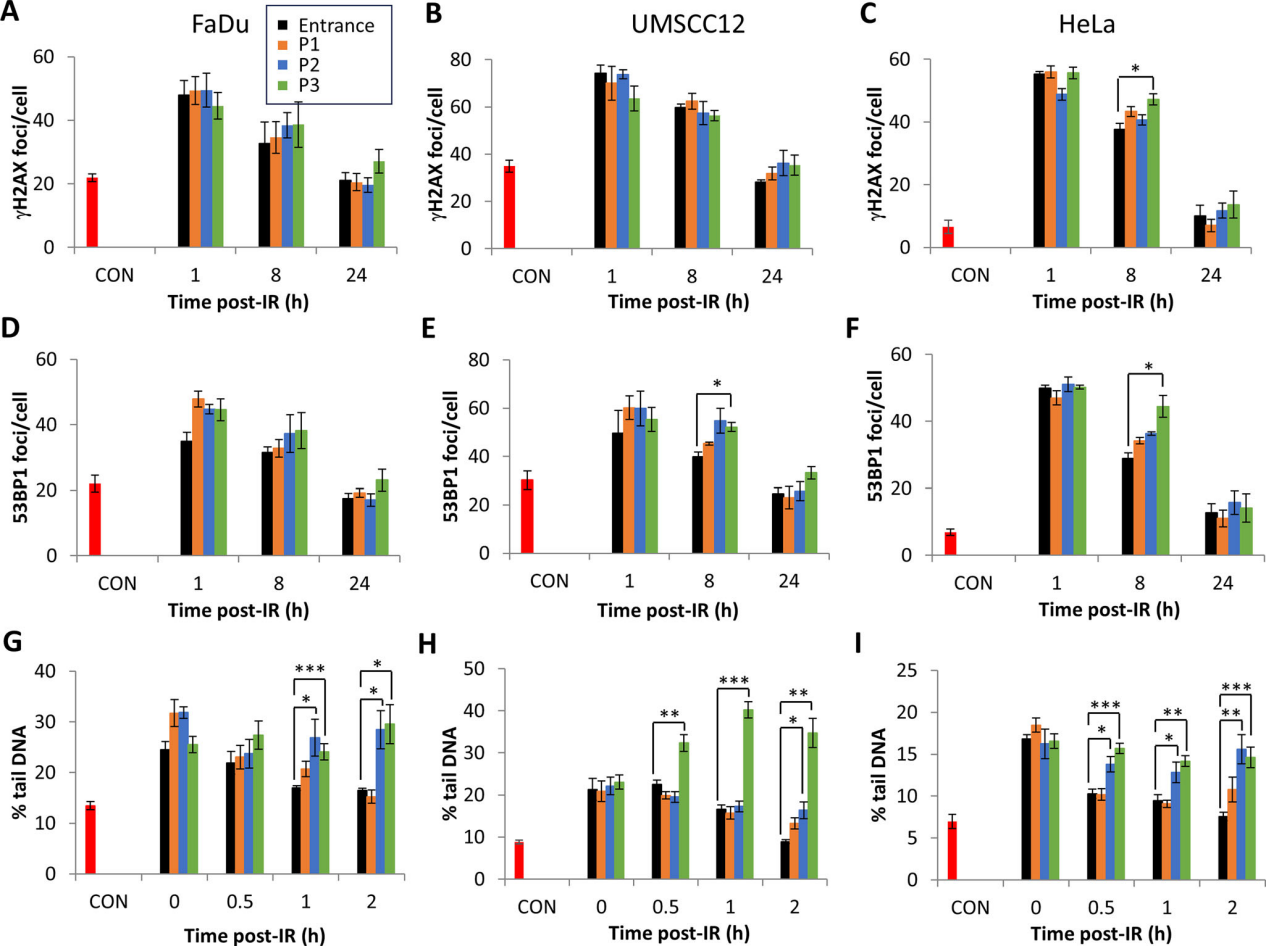
Protons with increasing LET generate DNA SSB/ALSs that are more persistent. **A**, **D** and **G** FaDu, **B**, **E** and **H**) UMSCC12 and **C**, **F** and **I** HeLa cells were irradiated (4 Gy) with entrance dose 28 MeV protons or the different positions across a pristine Bragg peak with increasing LET. Cells were then incubated at the respective time points to enable DNA repair. **A–C** γH2AX or **D–F** 53BP1 foci were analysed by immunofluorescence microscopy. **G–I** SSBs and ALSs were measured by the alkaline comet assay. Shown is the mean % tail DNA ± S.E. *p < 0.05, **p < 0.02, ***p < 0.001 as analysed by a one sample *t*-test.

We analysed the levels and repair of DSBs and SSBs/alkali-labile sites (ALSs) in HeLa and HNSCC cell lines using γH2AX and 53BP1 foci (as a surrogate marker of DSBs) and alkaline comet assays (for SSBs/ALSs) following PBT at increasing LET. We did not observe any significant increases in γH2AX foci formation at 1 h post-irradiation in HeLa and HNSCC cells comparing entrance dose protons and the different positions across a pristine Bragg peak (Fig. 2A–C and Supplementary Fig. S3 and S4). Analysing the kinetics of repair post-irradiation, there were no significant difference in γH2AX foci levels at 8-24 h post-irradiation as a function of LET (apart from 8 h post-irradiation in HeLa cells at the highest LET). There appeared to be some persistence in γH2AX foci particularly at 24 h using the highest LET generated at the Bragg peak, though this was not statistically significant in any of the cell lines. Similar observations were seen with 53BP1 foci as a DSB marker (Fig. 2D-F and Supplementary Fig. S5-6). An apparent delay in resolving of 53BP1 foci, and therefore of DSBs, was observed with protons with increasing LET at 8 h and 24 h post-irradiation. However, this was only statistically significant in UMSCC12 and HeLa cells at 8 h post-irradiation with protons at the Bragg peak with the highest LET (Fig. 2E, F and Supplementary Fig. S6). In general, this data demonstrates that there are no significant increases in the levels and/or delays in the repair of DSBs relative to PBT LET.

Using alkaline comet assays, we observed that there were no statistically significant increases in SSBs/ALSs immediately post-irradiation comparing entrance dose protons and the different positions across a pristine Bragg peak with increasing LET (Fig. 2G–I and Supplementary Fig. S7A, B). However, there were delays in the repair of SSBs/ALSs particularly with protons delivered close to and at the Bragg peak (5.4 and 7.8 KeV/µm), and which is statistically significant at the 1 and 2 h time points in all three cell lines. This data demonstrates that relatively high-LET protons can induce CDD sites that are SSB/ALS-associated, which persist and require longer time for efficient repair.

### CDD persistence is observed in cells with increasing proton LET

**Fig. 3:**
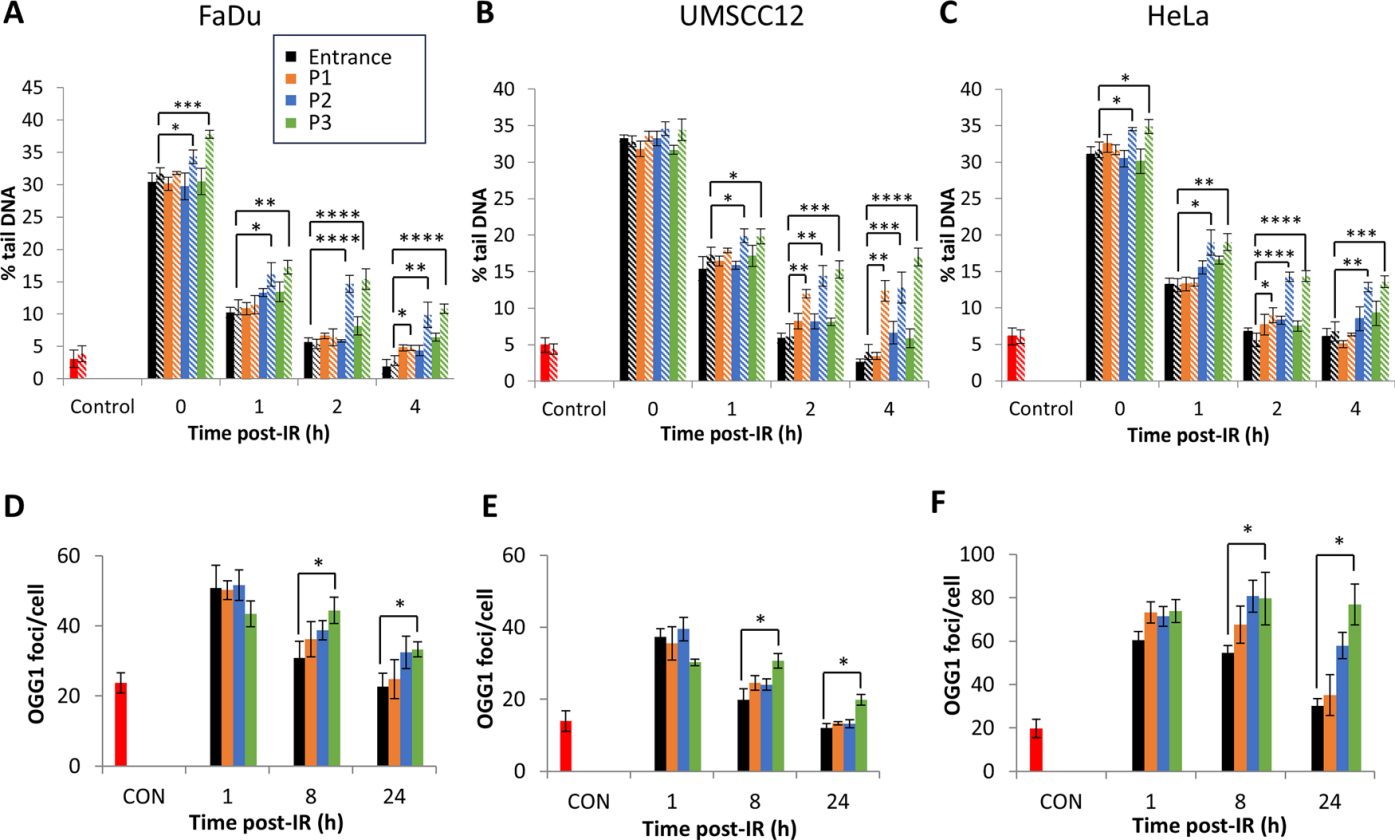
Protons with increasing LET generate increased levels of more persistent CDD. **A** and **D** FaDu, **B** and **E** UMSCC12 and **C** and **F** HeLa cells were irradiated (4 Gy) with entrance dose 28 MeV protons or the different positions across a pristine Bragg peak with increasing LET. Cells were then incubated for the respective time points to enable DNA repair. **A–C** DNA damage was measured at various time points post-IR by the enzyme modified neutral comet assay following incubation in the absence (revealing DSBs; solid bars) or presence (revealing CDD; hatched bars) of the recombinant enzymes APE1, NTH1 and OGG1. Shown is the mean % tail DNA ± S.D. *p < 0.05, **p < 0.05, ***p < 0.002, ****p < 0.0005 as analysed by a one sample *t*-test. **D–F** Alternatively, OGG1 foci were analysed by immunostaining. Shown is the mean OGG1 foci per nuclei ±S.D. *p < 0.01 as analysed by a one sample *t*-test.

We recently developed an enzyme-modified neutral comet assay to measure CDD (using the enzymes NTH1, OGG1 and APE1 to incise residual oxidised pyrimidines, oxidised purines and ALS, respectively), and also demonstrated that OGG1 can act as a surrogate marker to analyse the repair of non-DSB-associated CDD sites generated by PBT [15]. Using these approaches, we firstly demonstrated that on analysis of neutral comet assay data in the absence of enzyme modification (which reveals the direct levels and repair of DSBs), and similar to γH2AX/53BP1 foci analysis, that the immediate levels of DSBs induced comparing entrance dose protons and the different positions across a pristine Bragg peak does not change in the cell lines (Fig. 3A–C; compare solid black, orange, blue and green bars; Tables 3–5 and Supplementary Fig. S8A, B). However, the repair of the DSB damage was notably delayed particularly at 2–4 h post-irradiation using the highest LET generated at the Bragg peak (7.8 keV/µm), and which is statistically significant compared to DSB levels seen using entrance dose protons (Tables 3–5). Using enzyme modification to reveal CDD sites (residual oxidative DNA base damage and abasic sites proximal to DSBs), we observe that there were statistically significant increases in CDD that persists for 1–4 h post-irradiation with protons delivered close to and at the Bragg peak (5.4 and 7.8 KeV/µm, respectively; Fig. 3A–C, compare hatched black, orange, blue and green bars and Supplementary Fig. S8C, D). On analysis of OGG1 foci as a surrogate marker of CDD, we observed increased levels and persistence of OGG1 foci particularly at 8–24 h post-irradiation as a function of increasing LET (Fig. 3D–F). Statistically significant increased levels of OGG1 foci at 8 and 24 h post-irradiation in cells treated with high-LET protons generated at the Bragg peak compared to entrance dose protons were observed. Collectively, the data acquired from enzyme-modified neutral comet assays and OGG1 foci are consistent in demonstrating the formation of CDD with protons delivered at the Bragg peak with increased LET, and that the damage is significantly more persistent in taking a longer time to repair.

**Table 3:** Statistical analysis of DSB repair kinetics acquired from neutral comet assay data from FaDu cells treated with protons of increasing LET.

Time post-IR (h)	P1	P2	P3
0	0.77	0.65	0.97
1	0.46	0.01	0.03
2	0.11	0.69	0.05
4	0.02	0.04	0.003

Statistical analysis performed using a one sample *t*-test comparing DSB levels from the above conditions versus entrance dose protons.

**Table 4:** Statistical analysis of DSB repair kinetics acquired from neutral comet assay data from UMSCC12 cells treated with protons of increasing LET.

Time post-IR (h)	P1	P2	P3
0	0.45	0.99	0.02
1	0.16	0.66	0.24
2	0.84	0.03	0.01
4	0.10	0.01	0.01

Statistical analysis performed using a one sample *t*-test comparing DSB levels from the above conditions versus entrance dose protons.

**Table 5:** Statistical analysis of DSB repair kinetics acquired from neutral comet assay data from HeLa cells treated with protons of increasing LET.

Time post-IR (h)	P1	P2	P3
0	0.18	0.52	0.44
1	0.93	0.03	0.004
2	0.34	0.02	0.21
4	0.19	0.10	0.05

Statistical analysis performed using a one sample *t*-test comparing DSB levels from the above conditions versus entrance dose protons.

### Increased chromosomal aberrations are evident in cells with increasing proton LET

**Fig. 4:**
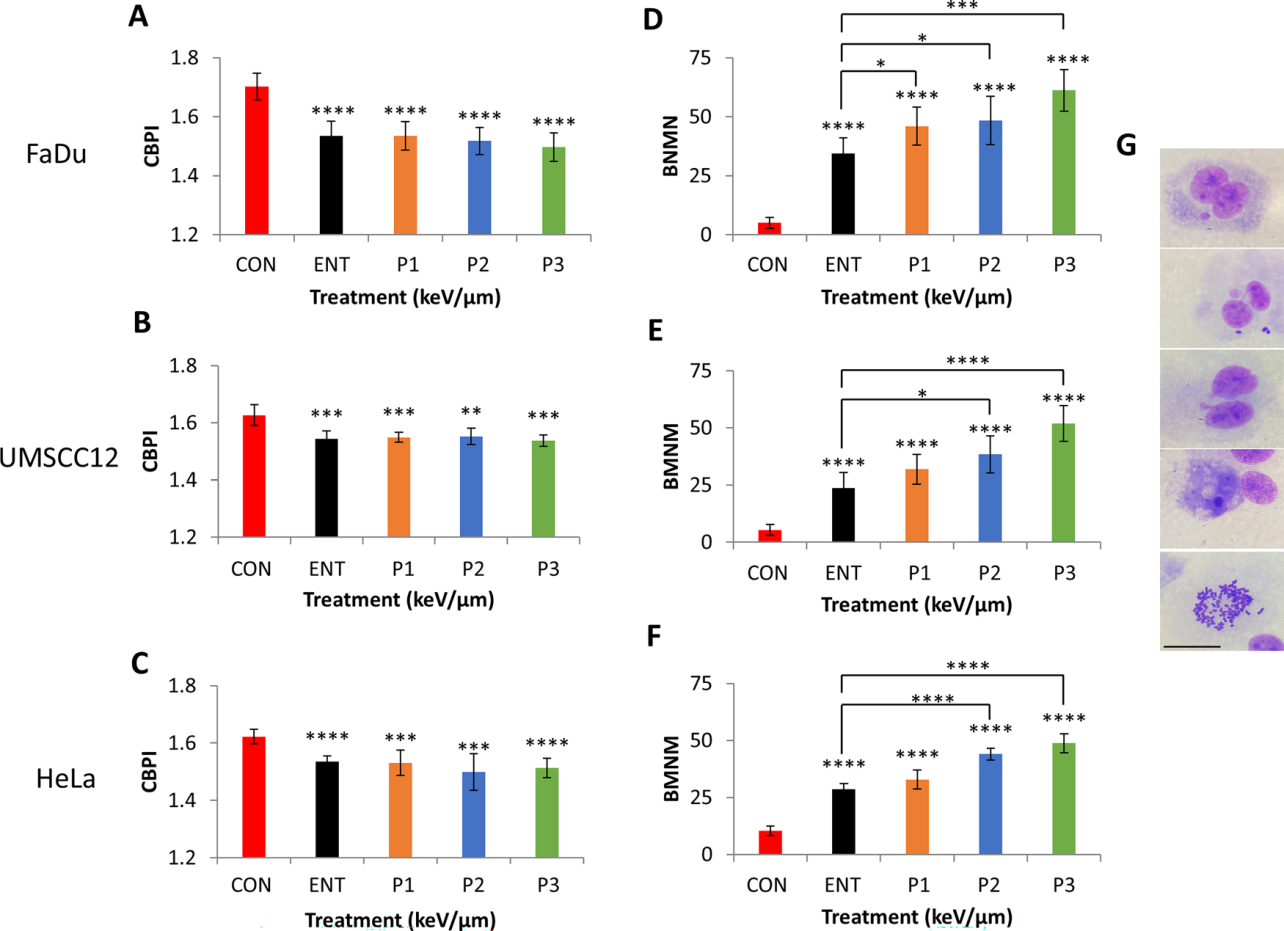
Protons with increasing LET induce chromosomal aberrations. **A** and **D** FaDu, **B** and **E** UMSCC12, and **C** and **F** HeLa were irradiated with entrance dose 28 MeV protons or the different positions across a pristine Bragg peak with increasing LET. Proliferation index and micronuclei frequency was then scored by the cytokinesis-block micronuclei assay. Shown is **A–C** mean CBPI ± S.E. and **D–F** micronuclei frequency calculated as the mean binucleated with micronuclei scored in 1000 binucleated cells±S.E. *p < 0.05, **p < 0.01, ***p < 0.005, ****p < 0.001 as analysed by a one sample *t*-test. **G** Representative images (from top *t*o bottom) of FaDu binucleated with micronuclei cell, nuclear bridge with micronuclei, nuclear buds, necrotic cell and mitotic cell, respectively.

We analysed the effect of PBT with increasing LET on proliferative ability and frequency of chromosomal abnormalities in HeLa and HNSCC cells using the cytokinesis-block micronucleus assay. Cytokinesis-block proliferation index (CBPI) was expectedly significantly reduced in all cell lines after PBT treatment compared to the unirradiated control, however no statistically significant differences comparing entrance dose protons and the different positions across a pristine Bragg peak were observed (Fig. 4A–C). On analysis of micronuclei frequency, this was found to be significantly elevated following PBT irradiation compared to the unirradiated control cells. Additionally, there were statistically significant increases in micronuclei at the different positions across a pristine Bragg peak with increasing LET relative to entrance dose protons (Fig. 4D-F). The highest micronuclei frequency was observed at the Bragg peak with the highest LET. Results on chromosome rearrangements, detected as nucleoplasmic bridges, and frequencies of nuclear buds (NBUDs) as a biomarker of amplified DNA elimination, are summarised (Supplementary Table S1), while apoptotic and necrotic cells were also scored (Supplementary Table S2). A significant increase in nucleoplasmic bridges and nuclear buds was observed in HeLa and UMSCC12 cells compared with the unirradiated control, across all the irradiation positions. Interestingly, only the incidence of nucleoplasmic bridges were increased in FaDu cells and only at positions proximal to the Bragg peak. Moreover, there was a marked increase in nuclear buds relative to LET in all cell lines, and which was statistically significant at the Bragg peak with the highest LET compared to entrance dose protons in HeLa and UMSCC12 cells. In terms of apoptotic cells, these expectedly increased post-irradiation in all cell lines compared to the unirradiated controls. In both HeLa and FaDu cells, increased apoptosis was statistically significant at 4.3–7.8 KeV/µm protons, while this was evident at only 7.8 keV/µm in UMSCC12 cells. Statistically significant increased levels of necrotic cells were observed in HeLa and UMSCC12 cells post-irradiation versus the unirradiated control, whilst this was not evident in FaDu cells. There was no evidence of increased apoptosis or necrosis as a function of LET in any of the cell lines. Mitotic index was generally in line with the CBPI results, where there was evidence of decreased mitosis post-irradiation versus the unirradiated control, although no significant differences were observed as a function of increasing LET.

Taken together, our data confirmed that protons of increasing LET at and around the Bragg peak cause increased chromosomal aberrations in HeLa and HNSCC cells.

## Discussion

Despite the dosimetric advantages of PBT through the precise delivery of the radiation dose to the tumour, there remains biological and clinical uncertainty with the treatment due to the increases in LET at and around the Bragg peak leading to an enhanced RBE. The RBE of 1.1 used in treatment planning has therefore been questioned [2, 3]. A major consequence of the increased LET is the enhanced density of ionisation events leading to the formation of CDD which represents a major challenge to the cellular DNA repair machinery and its persistence post-irradiation. CDD is therefore considered a major contributor to the enhanced RBE of PBT [1]. Despite this, there is still a need to perform systematic studies to investigate the radiobiological responses of well-characterised cell models to protons at and around the Bragg peak where the LET is the greatest, using stable beam lines.

In this study, we have shown using a 28 MeV pristine proton beam, that survival of HNSCC and HeLa cell lines decreases at the Bragg peak in comparison to the entrance dose, and that maximal radiosensitivity and therefore RBE of between 1.44–2.02 is achieved at the Bragg peak. This is supported by other studies that have demonstrated that there are differences in the RBE along the depth dose of the proton beam. For example, it has been shown using FaDu cells irradiated with protons at the proximal and distal SOBP (LET of 2.1 and 4.5 keV/μm, respectively) that there are increases in RBE of up to 1.4 at the highest LET [16]. Human fibroblasts and glioma cells irradiated along pristine and modulated 62 MeV proton beams showed RBE values of >3 in the distal region at the Bragg peak (equivalent to an LET of 22 keV/µm) [11]. Additionally, an RBE of 2.65 for fibrosarcoma HT-1080 cells whilst a much lower RBE of 1.51 for endothelial HMEC-1 cells were identified following irradiation at the distal edge of a 100 MeV proton pencil beam (LET = 14.47 keV/μm) [17].

Interestingly, we observed that HeLa cells generally revealed lower RBE values across the different irradiation positions compared to two HNSCC cells. This is not uncommon based on the literature. Indeed, different RBE values have been observed when comparing human Hep2 laryngeal cancer cells and V79 cells at various positions along the SOBP of a beam with an incident energy of 87 MeV (LET ranging from 5.3-28.8 keV/µm), and where the values differed from 1.46–2.3 (Hep2) and 1.23–1.78 [12]. When comparing Chinese hamster ovary, A549 human lung adenocarcinoma and T98 human glioma cells exposed to 71 MeV and 160 MeV protons at depths along the Bragg curve, it has also been shown that RBE values ranged from 0.89-2.40, but where A549 and T98 cells generally displayed higher biological responses [18]. Taken together, these data suggest that there is a contribution of the cell line origin and inherent radiosensitivity to the radiobiological response of protons with increasing LET.

Another factor influencing RBE is the structure and architecture of the cell model used. Irradiation of cells within a 3D structure could potentially show different responses to protons of increasing LET compared to 2D monolayers, mainly driven by oxygen levels and particularly the hypoxic core of the 3D structure, which could harbour radioresistance, at least to low-LET protons. However, it has been reported that there was no apparent difference in biological effectiveness of protons on EMT6 single cells versus V79 spheroids relative to LET [19]. These results are difficult to analyse given the two different models used, but may also be associated with the fact that the LET at the distal end of the proton beam ( ~ 9.5 keV/μm) was likely not high enough to establish any biological differences. Interestingly, an in vivo model of murine cervical spinal cord showed a relatively small (1.1–1.2) increase in RBE (calculated as acute toxicity and grade II paresis) following irradiation with high-LET protons (6.9 keV/μm) compared to entrance dose controls (1.2 keV/μm) [20].

Along with increased RBE, we demonstrate an association with increased levels and persistence of CDD at the Bragg peak, through both enzyme-modified neutral comet assays and the surrogate marker OGG1. Increased persistence of 53BP1 foci, as a marker of DSBs, has also been seen at the distal end of a spread-out Bragg peak using a 60 MeV proton beam [14]. Similarly, it has recently been shown that there is increased persistence of 53BP1 associated with larger foci size in FaDu cells following protons delivered at the distal end compared to entrance dose protons [21]. We also observed increased persistence of γH2AX/53BP1 foci at the Bragg peak distal end although these were not statistically significant compared to entrance dose protons. Similarly, the persistence of γH2AX foci in FaDu and A549 cells were observed not to be different with protons delivered at the proximal and distal end (LET of 2.1 and 4.5 keV/µm, respectively) [16]. Using a modulated 62-MeV proton beam with human melanoma, human breast adenocarcinoma and non-small lung cancer cells irradiated at different positions relative to the Bragg peak (corresponding to LET values of 2.2–19.3 keV/µm), increases in γH2AX foci have been observed although the kinetics of repair was not investigated [22]. Interestingly, high LET-like radiation tracks have been observed in CHO cells exposed to 70 MeV protons at the Bragg peak distal end (LET of 6.6 keV/μm) [23].

In contrast to DSBs, and through alkaline comet assays, we observed significant delays in the repair of SSBs/ALSs suggesting that the CDD induced under our conditions are more SSB/ALS in nature. This correlates with our previous data using a 60 MeV proton beam [24] but also where we subsequently identified that the single strand break binding protein PARP-1 is essential for the repair of CDD but also in maintaining cell survival in response to protons at relatively high-LET [25]. Furthermore, it has been shown using plasmid DNA and atomic force microscopy that DNA damage complexity increases with proton LET particularly at the Bragg peak distal end that induces DNA fragmentation [26]. Additionally, we also demonstrate significant increases in micronuclei frequency as a function of LET, and which are highest at the Bragg peak. This is consistent with the observed increases in more persistent CDD, and which are likely to be the driving factors in promoting chromosomal aberrations through either misrepair or following DNA replication. Indeed, it has been previously reported that there are increases in chromosomal aberrations and which take a longer time to resolve in peripheral blood lymphocytes and Cal51 cells following irradiation with Bragg peak protons (LET of 1.4 keV/µm) compared to an unmodified 150 MeV protons beam (LET of 0.57 keV/µm) [27]. This is supported by data in CHO cells demonstrating an increase in chromosomal aberrations as a function of radiation of increasing LET [28]. Given that CDD is known to contribute to the cell-killing effects of PBT and other ionising radiation sources, it is surprising that we still have limited understanding of the molecular and cellular response to this type of DNA damage [6, 10], and which should be the focus of future research.

## Materials And Methods

### Antibodies

The following antibodies were used: OGG1 (NB100-106; Bio-Techne Ltd, Abingdon, UK), γH2AX (05-636; Merck-Millipore, Watford, UK), 53BP1 (A300-272A; Bethyl Labs, Montgomery, Texas, USA). Goat anti-mouse Alexa Fluor 555 (A21422) or goat anti-rabbit Alexa Fluor 488 (A11008) secondary antibodies for immunofluorescence microscopy were from Thermo Fisher Scientific (Massachusetts, USA).

### Cell culture and irradiation sources

Laryngeal squamous cell carcinoma (UMSCC12) and HeLa cells were kindly provided by Prof T. Carey (University of Michigan, USA) and Prof G. Dianov (University of Oxford, UK), respectively. Hypopharyngeal squamous cell carcinoma cells (FaDu) were acquired from ATCC (Teddington, UK). All cell lines were authenticated in our laboratory by STR profiling, and were routinely cultured as monolayers in 5% CO_2_ at 37 °C. Cells were cultured in Dulbecco’s Modified Eagle Medium supplemented with 10% foetal bovine serum, 2 mM L-glutamine, 1× penicillin-streptomycin and 1× non-essential amino acids, except for FaDu cells, which were cultured in Modified Eagle Medium (MEM). For irradiations, cells grown in 35 mm dishes were exposed to a horizontal, passive-scattered proton beam line of 28 MeV maximal energy at a dose rate of ~5 Gy/min. Degraders were used to precisely position the cells at different positions relative to the Bragg peak. The track-averaged LET values were calculated using Geant4 toolkit, as previously described [29] (see also Supplementary Methods).

### Clonogenic assays

Clonogenic assays were performed as previously described [30]. Plating efficiencies for the cells were as followed: HeLa (~ 40%), FaDu and UMSCC12 (~20%). Colonies (>50 cells) were counted using the GelCount colony analyser (Oxford Optronics, Oxford, UK), which was optimised for all cell lines, based on inclusion of distinct colonies of specific size and intensity, although the same settings were used across the various treatments. Relative colony formation (surviving fraction) was expressed as colonies per treatment level versus colonies that appeared in the untreated control.

### Alkaline comet assay

The alkaline comet assay for measurement of DNA SSBs and ALSs was performed as previously described [30]. Briefly, cells following irradiation were trypsinised, diluted to 1 × 10^5^ cells/ml and 250 μl of the cell suspension was embedded in 1% low-melting-point agarose on a precoated microscope slide (Bio-Rad, Hemel Hempstead, UK). Slides were incubated for up to 4 h in a humidified chamber at 37 °C to allow for DNA repair, prior to lysis in buffer containing 2.5 M NaCl, 100 mM EDTA, 10 mM Tris-HCl pH 10.5, 1% (v/v) DMSO and 1% (v/v) Triton X-100 for 1 h at 4 °C. Slides were then incubated in the dark for 30 min in cold electrophoresis buffer (300 mM NaOH, 1 mM EDTA, 1% (v/v) DMSO, pH 13) to allow the DNA to unwind, prior to electrophoresis at 25 V, 300 mA for 25 min. Slides were neutralised with three 5 min washes of 0.5 M Tris-HCl (pH 8.0) and allowed to air dry overnight. Following rehydration for 30 min in water (pH 8.0), the DNA was stained for 30 min with SYBR Gold (Life Technologies, Paisley, UK) diluted 1:10,000 in water (pH 8.0) and again air dried overnight. Cells (50 per slide, in duplicate) were analysed from the dried slides using the Komet 6.0 image analysis software (Andor Technology, Belfast, Northern Ireland) and % tail DNA values averaged from at least three independent, biological experiments.

### Enzyme-modified neutral comet assay

Detection of DSBs and CDD was achieved using the enzyme-modified neutral comet assay, as recently described [15]. In brief, and following cell lysis after proton-induced DNA damage and repair, slides were washed three times with enzyme reaction buffer (40 mM HEPES-KOH, 100 mM KCl, 0.5 mM EDTA and 0.2 mg/ml BSA, pH 8.0), and then incubated with either buffer alone (mock treated; revealing levels of DNA DSBs) or with buffer containing 5 pmol OGG1, 6 pmol NTH1 and 0.6 pmol APE1 (enzyme treated; revealing levels of DNA DSBs plus CDD) for 1 h at 37 °C in a humidified chamber. Slides were subsequently placed in cold electrophoresis buffer (1× TBE buffer (pH 8.3)) in the dark for 25 min to allow the DNA to unwind, prior to electrophoresis at 25 V, ~20 mA for 25 min. Slides were washed three times with 1 × PBS, dried and processed as described in the alkaline comet assay method.

### Immunofluorescence

Measurement of DNA repair protein foci (γH2AX, 53BP1 and OGG1) were examined as previously described [30]. In brief, cells were grown on 13 mm coverslips until ~70–80% confluent, irradiated at 4 Gy and incubated for up to 24 h in 5% CO_2_ at 37 °C to allow for DNA repair. Cells were washed with PBS at room temperature for 5 min, before being fixed using 4% paraformaldehyde for 10 min. Cells were permeabilised with 0.2% Triton X-100 in PBS for 10 min, washed three times with 0.1% Tween-20 in PBS for 10 min, and blocked to avoid non-specific staining via incubation with 2% BSA in PBS for 30 min at room temperature on a rocking platform. γH2AX/53BP1/OGG1 antibodies in 2% BSA were subsequently added, and coverslips incubated overnight at 4 °C. Following three washes with PBS, coverslips were incubated with either goat anti-mouse Alexa Fluor 555 or goat anti-rabbit Alexa Fluor 488 secondary antibodies in 2% BSA for 1 h at room temperature in the dark. Finally, samples were washed with PBS for 10 min on a rocking platform and mounted on a microscope slide using Fluoroshield containing DAPI (Sigma-Aldrich, Gillingham, UK). Cells were examined using an Olympus BX53 fluorescent microscope with a CAM-ORCA-FLASH4.0LTPlus Digital Hamamatsu sCMOS camera. CellSens Dimension Expandable Imaging software was used to capture images (~20 images/cell line/antibody).

### Cytokinesis-block micronucleus assay

The assessment of genotoxicity was performed as previously reported [31]. Briefly, cells were exposed to PBT (2 Gy) and cytochalasin B (5 µg/ml; Merck Life Science, Gillingham, UK) was added to block the first cytokinesis process at a specific cell line-dependent timepoint (for UMSCC12, HeLa and FaDu at 16 h, 17 h and 18 h, respectively). Cells were harvested after 24 h, then treated with a hypotonic solution (75 mM KCl) for 3 min, prefixed in 3:5 methanol:acetic acid, washed once with methanol, and subsequently fixed twice with a 6:1 methanol:acetic acid fixative solution. Finally, the cell solution was dropped onto cold glass microscope slides. The staining procedure was performed by immersing the air-dried slides in a 2% Giemsa solution in Sorensen’s buffer (pH 6.8). Binucleated cells (2000) were examined for each experimental point in a blind mode (1000 from each independent culture replicate) using a Nikon Eclipse 800 optical microscope (×400 magnification). The scoring criteria adopted by Fenech [32] was followed for each endpoint. Briefly, we evaluated the binucleate micronucleated cells frequency as number of binucleate cells containing one or more micronuclei per 1000 binucleated cells. Moreover, 500 cells were scored to evaluate the percentage of mono-, bi- and multinucleated cells, and the CBPI was calculated as an index of cytostasis by comparing values in the treated and control cultures. The CBPI indicates the average number of cell cycles per cell during the period of exposure to Cytochalasin B and may be used to calculate cell proliferation. Finally, other damage events were scored in once-divided bi-nucleated cells per 1000 cells, namely: (i) nucleoplasmic bridges, a biomarker of DNA misrepair and/or telomere end-fusions, and (ii) nuclear buds, a biomarker of elimination of amplified DNA and/or DNA repair complexes. The number of apoptotic, necrotic, and mitotic cells were also evaluated.

### Statistical analysis

All experiments were performed in at least triplicate as separate, independent, biological experiments. Statistical analysis of cytokinesis-block micronucleus assay results was performed using a one-way ANOVA. Statistical analysis of clonogenic survival data was performed using the CFAssay for R package [33], which uses the linear-quadratic model to compare different treatment responses across increasing radiation doses. Statistical analysis of DNA DSB damage quantified through neutral comet assays was performed using either a one-sample or two-sample *t* test.

## Supplementary information

Supplementary Data

## Data Availability

Source data are provided within this paper and the Supplementary Data. Any other data will be made available from the corresponding author upon reasonable request.
