# Supplementary material for: Radiobiological characterisation of a 28 MeV proton beam delivered by the MC-40 cyclotron
Source: Cell Death Discov. 2025 Jul 21;11:334. doi: 10.1038/s41420-025-02635-1 (PMC12280003; doi:10.1038/s41420-025-02635-1)
Supplement: Supplementary file 1 — Supplementary Data [file 41420_2025_2635_MOESM1_ESM.docx]

**SUPPLEMENTARY METHODS**

Monte Carlo simulations were performed using Geant4 v.11.2.2 to investigate four proton energy degradation scenarios (four positions): no absorber; a 3 mm PMMA block with a 0.5 mm polypropylene shim, a 4 mm PMMA block, and a 4 mm PMMA block with a 0.5 mm polypropylene shim. The initial proton energy was (27.90 ± 0.05) MeV, determined via Advanced Markus chamber dosimetry and characterised by a Gaussian profile. Beam radial divergence was determined to be 0.750 mm using EBT3 film dosimetry. Proton fluence was adjusted to ensure 30,000 events within the 10 µm-thick cellular layer, therefore limiting statistical uncertainty to approximately 0.5%.

The geometry of the MC40 cyclotron facility in Birmingham was modelled in Geant4 to determine the proton energy and LET values at the cell layer. The cellular target was represented as a water-equivalent cylinder 10 µm in height and 35 mm in diameter, corresponding to the dimensions of standard 35 mm tissue culture dishes. Track-averaged LET values were computed stepwise by recording energy deposition and step length at each simulation step, as per standard Monte Carlo particle-tracking methodology. LET was computed as the sum of deposited energies divided by the total track length within the scoring volume, with contributions from both primary and secondary protons included. LET was also calculated using the unrestricted electronic stopping power via Geant4 *ComputeElectronicDEDX()* function for benchmarking purposes.

To interpret the four scenarios of energy degradation, a reference Bragg peak in a simulated water phantom was generated using 27.9 MeV protons after passing through the beamline components as well as a 68 mm air gap and the cell dish bottom. This additional modelling step replicates experimental conditions and results in a lower mean energy of 21.44 MeV, reduced from the initial proton energy of 27.9 MeV due to interactions with beamline elements. The mean energy of protons reaching the cell layer in each case was used to indicate their approximate position along this curve. While the Bragg peak provides a useful visual reference, the actual energy and LET distributions in the simulations differ from those in water alone, as protons pass through plastic degraders and dish material before reaching the cells. These materials affect energy loss and scattering, so the plotted points reflect only the equivalent proton energy, not identical transport conditions. **A combined depth-dose and LET profile is shown in Supplementary Fig. S1A.**

Energy and dose scoring included all protons and secondary particles, including delta rays below Geant4’s standard production thresholds. Data analysis and visualisation, including proton energy spectra and per-step energy deposition distributions, were conducted using ROOT v.6.32.06.

**SUPPLEMENTARY DATA**

**
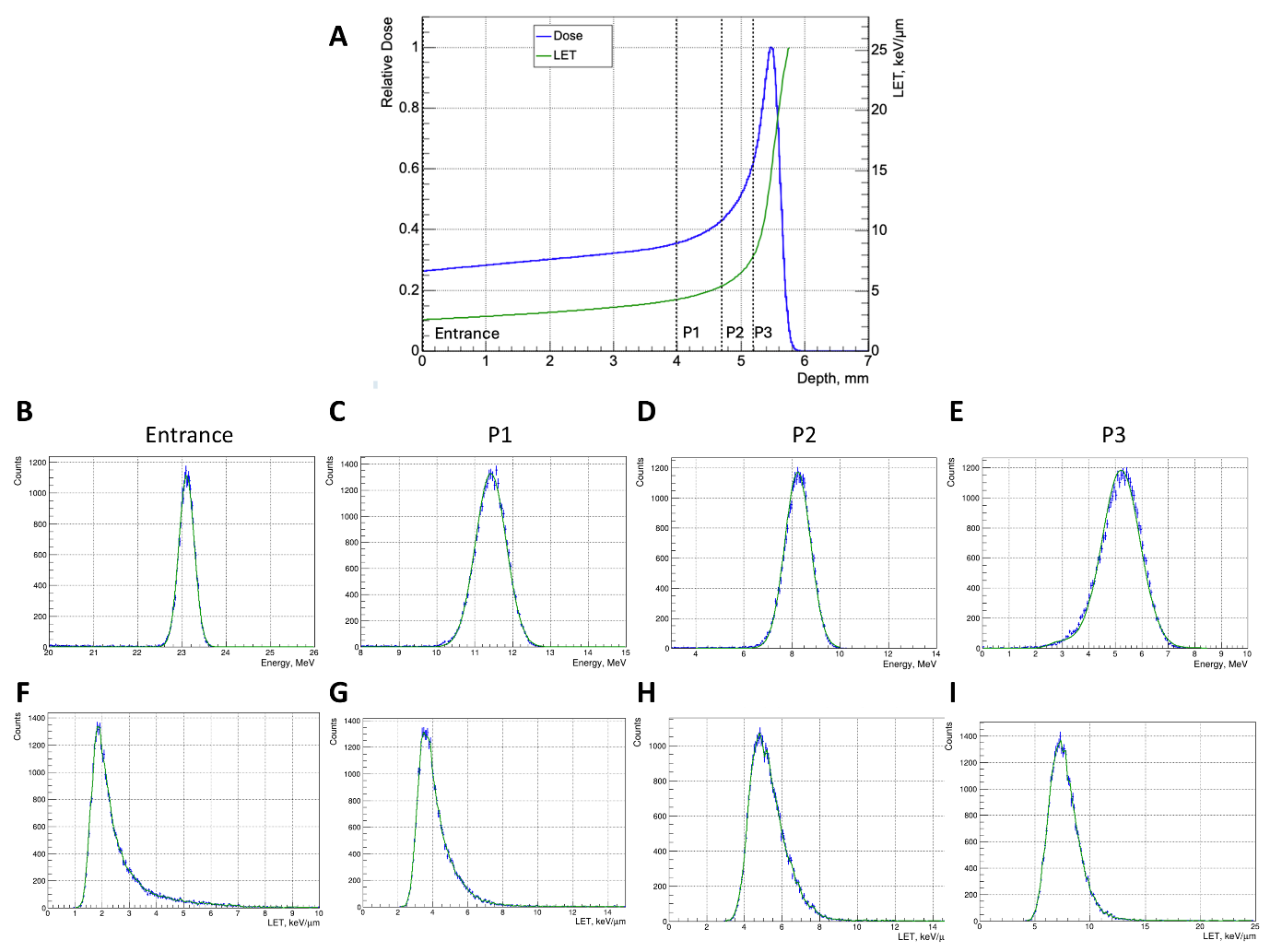
**

**Supplementary Figure 1. Simulated depth dose, LET distribution, energy and LET spectra from the MC-40 cyclotron delivered at different positions relative to the Bragg peak.** (A) Depth dose (blue) and mean linear energy transfer (LET, green) profiles for a 27.9 MeV proton beam in water, simulated using Geant4. The dose curve is normalised to unity at the Bragg peak. LET increases with depth, peaking near the Bragg peak and rapidly declining beyond it. Vertical dashed lines mark the approximate depths corresponding to the experimental irradiation positions: entrance (no absorber), P1 (3 mm PMMA + 0.5 mm polypropylene), P2 (4 mm PMMA), and P3 (4.5 mm PMMA + 0.5 mm polypropylene). (B-E) Energy spectra and (F-I) LET spectra calculated using Geant4 modelling.

**
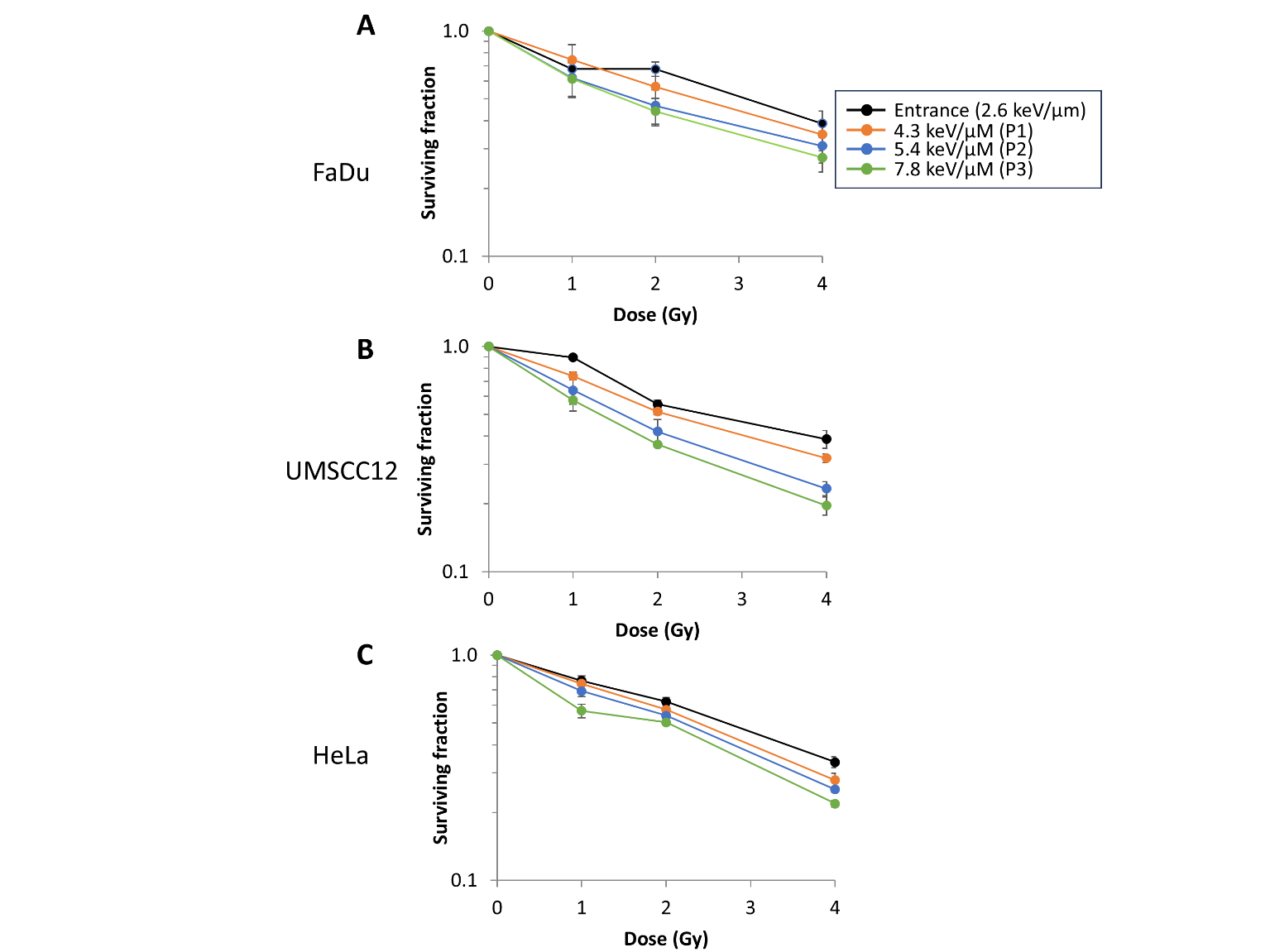
**

**Supplementary Figure 2. Protons with increasing LET lead to enhanced RBE in HeLa and HNSCC cells.** (A) FaDu, (B) UMSCC12, and (C) HeLa cells were irradiated with entrance dose 28 MeV protons or the different positions across a pristine Bragg peak with increasing LET. Clonogenic survival of cells was then analysed from three biologically independent experiments. Shown is the mean surviving fraction±S.E. complete with straight line fit.

**
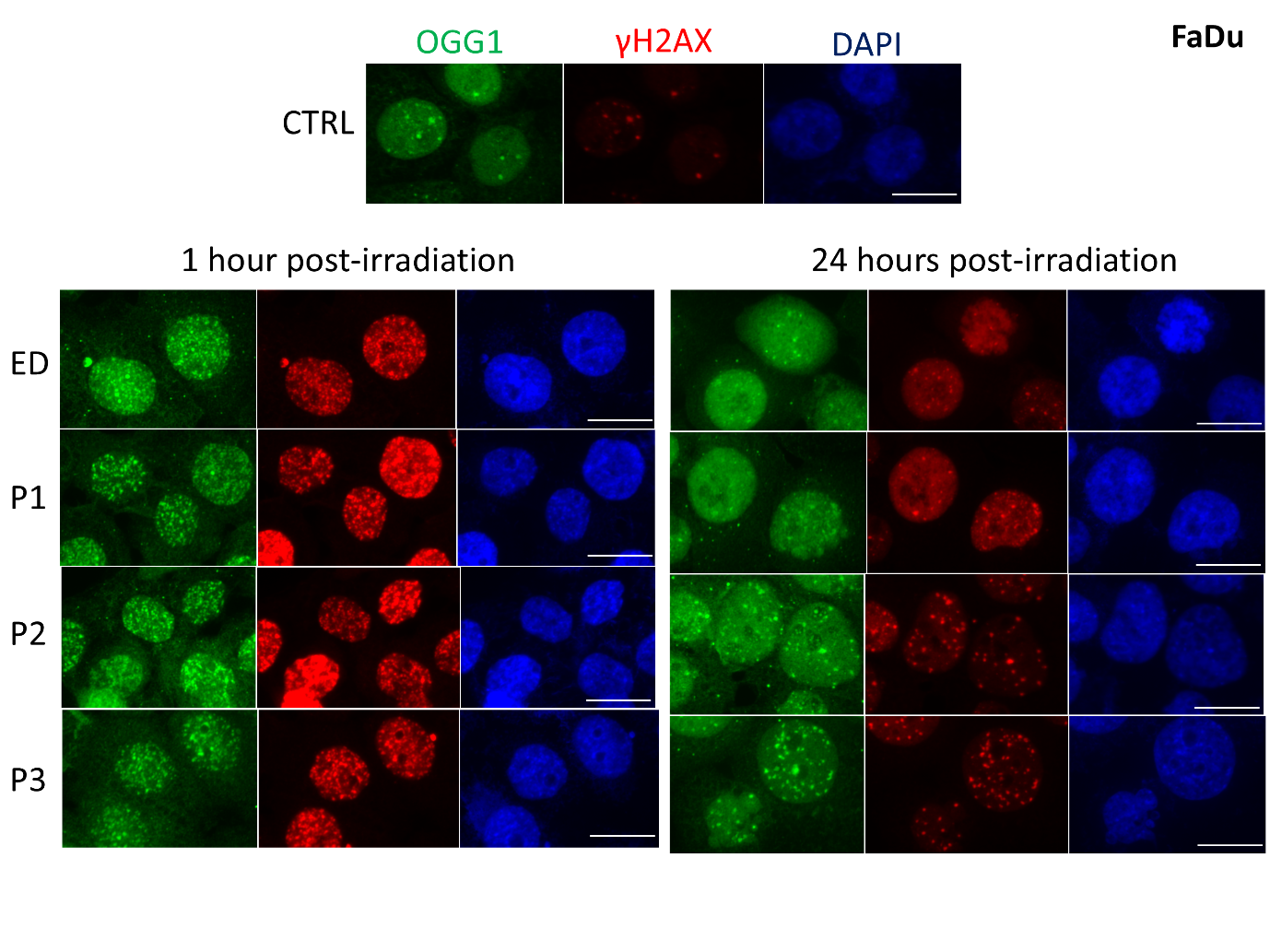
**

**Supplementary Figure 3. Protons with increasing LET lead to generation of persistent DNA damage revealed by OGG1 foci in FaDu cells.** Cells were irradiated (4 Gy) with entrance dose 28 MeV protons or the different positions across a pristine Bragg peak with increasing LET. Cells were then incubated at the respective time points to enable DNA repair, and γH2AX and OGG1 foci were analysed by immunofluorescence microscopy. Respective images of unirradiated cells (CTRL), and those at 1 and 24 hours post-irradiation, are shown.

**
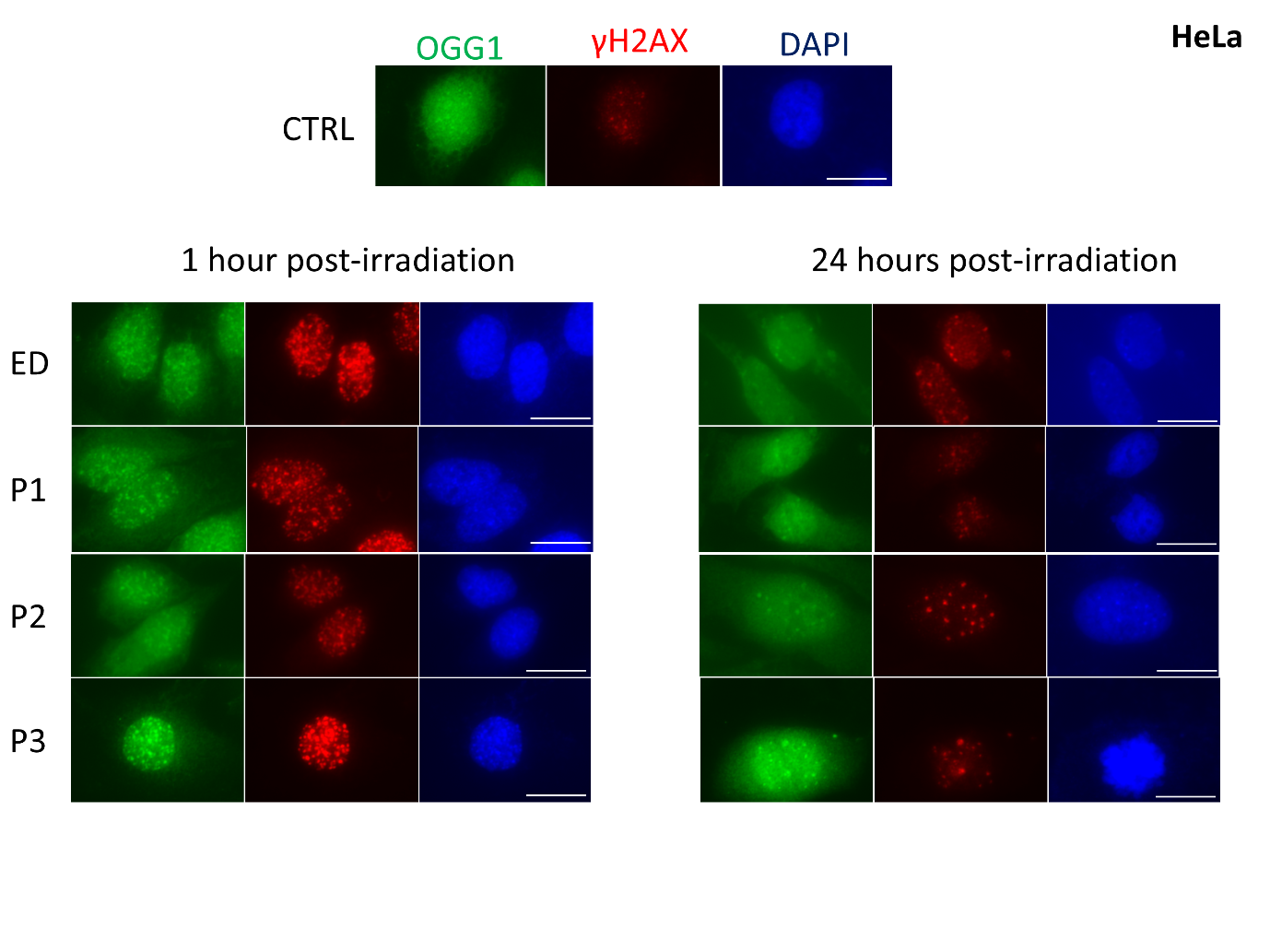
**

**Supplementary Figure 4. Protons with increasing LET lead to generation of persistent DNA damage revealed by OGG1 foci in HeLa cells.** Cells were irradiated (4 Gy) with entrance dose 28 MeV protons or the different positions across a pristine Bragg peak with increasing LET. Cells were then incubated at the respective time points to enable DNA repair, and γH2AX and OGG1 foci were analysed by immunofluorescence microscopy. Respective images of unirradiated cells (CTRL), and those at 1 and 24 hours post-irradiation, are shown.

**
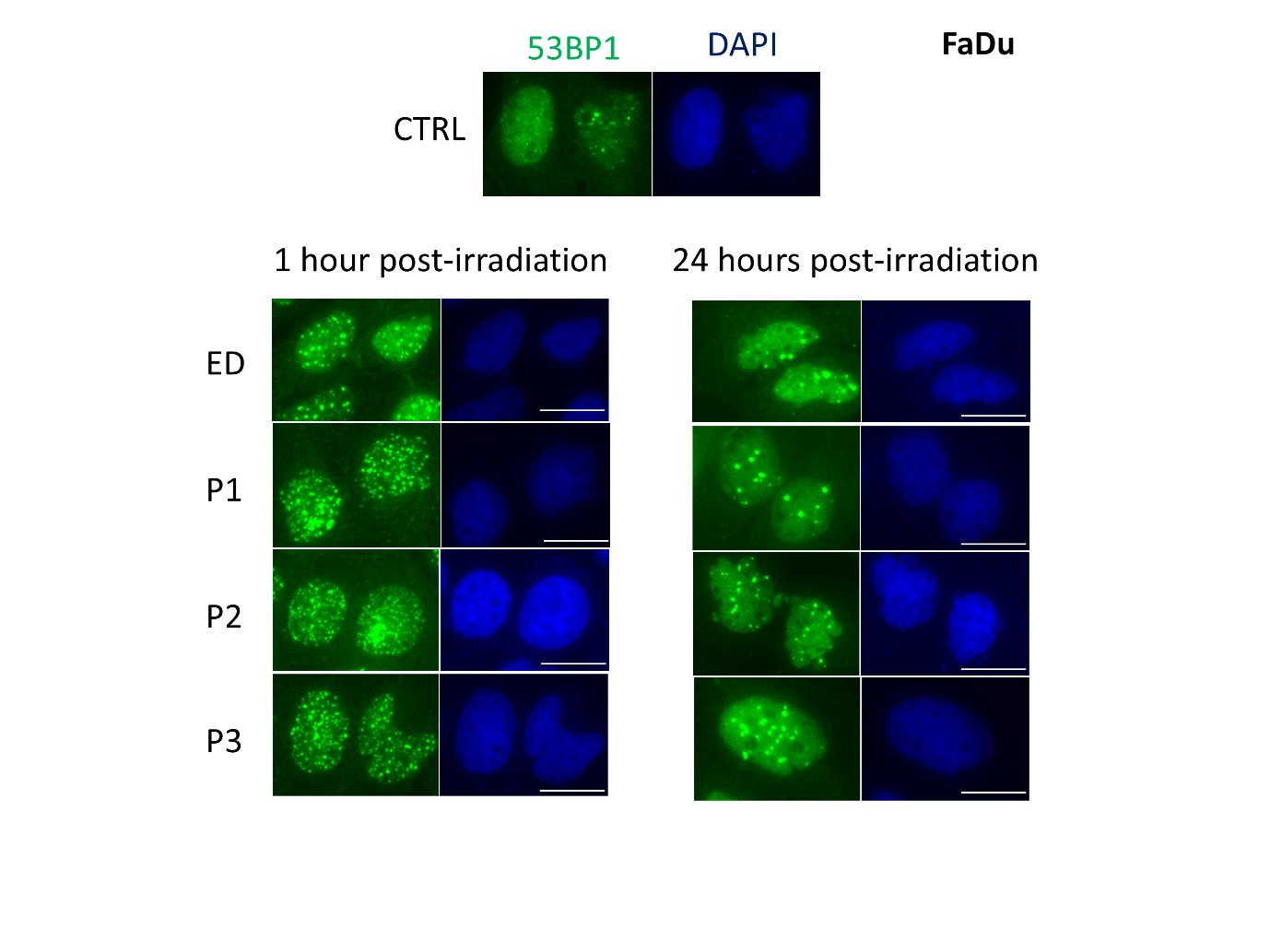
**

**Supplementary Figure 5. Protons with increasing LET do not lead to generation of persistent DNA DSBs in FaDu cells.** Cells were irradiated (4 Gy) with entrance dose 28 MeV protons or the different positions across a pristine Bragg peak with increasing LET. Cells were then incubated at the respective time points to enable DNA repair, and 53BP1 foci were analysed by immunofluorescence microscopy. Respective images of unirradiated cells (CTRL), and those at 1 and 24 hours post-irradiation, are shown.

**
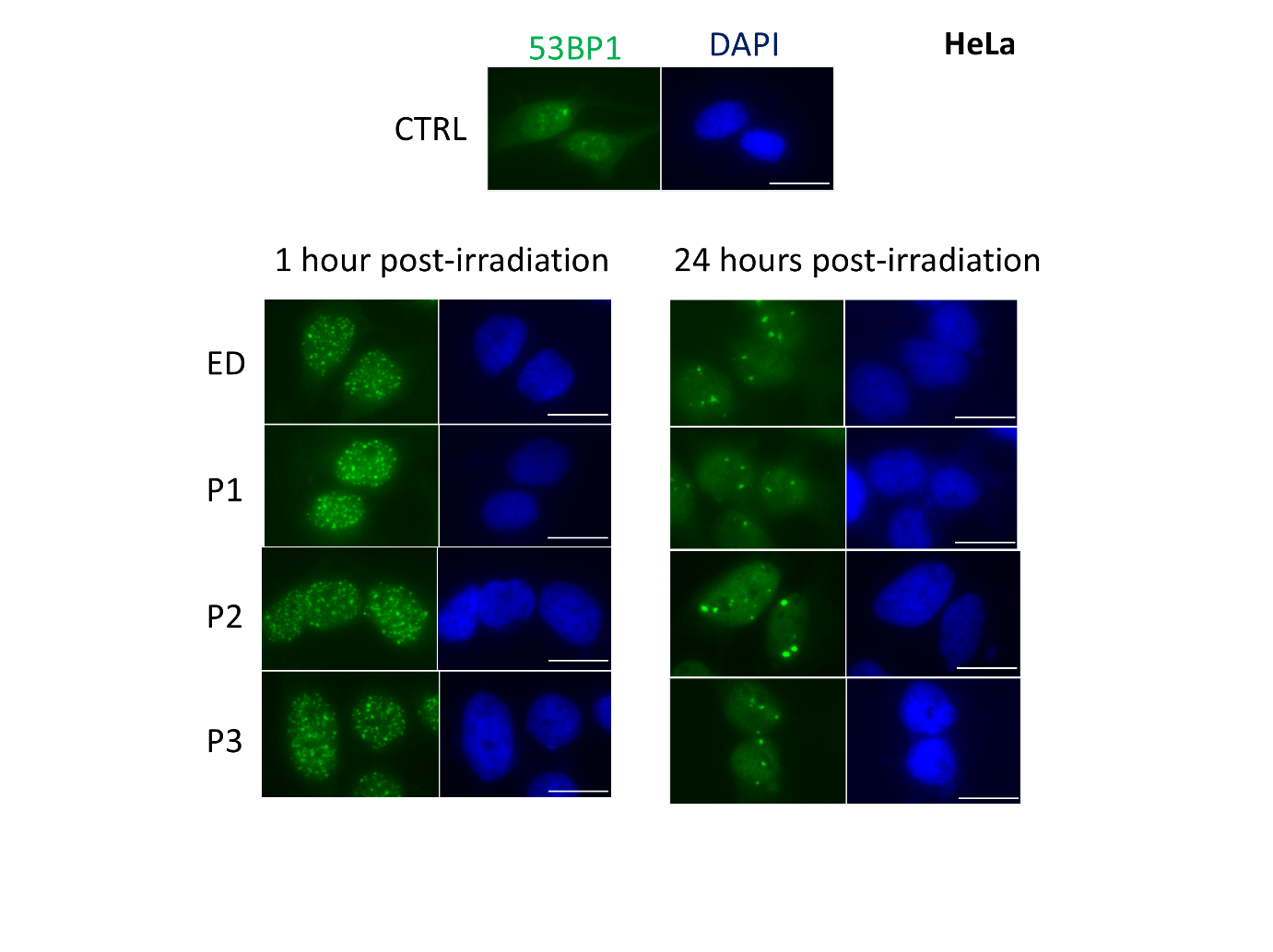
**

**Supplementary Figure 6. Protons with increasing LET do not lead to generation of persistent DNA DSBs in HeLa cells.** Cells were irradiated (4 Gy) with entrance dose 28 MeV protons or the different positions across a pristine Bragg peak with increasing LET. Cells were then incubated at the respective time points to enable DNA repair, and 53BP1 foci were analysed by immunofluorescence microscopy. Respective images of unirradiated cells (CTRL), and those at 1 and 24 hours post-irradiation, are shown.

**
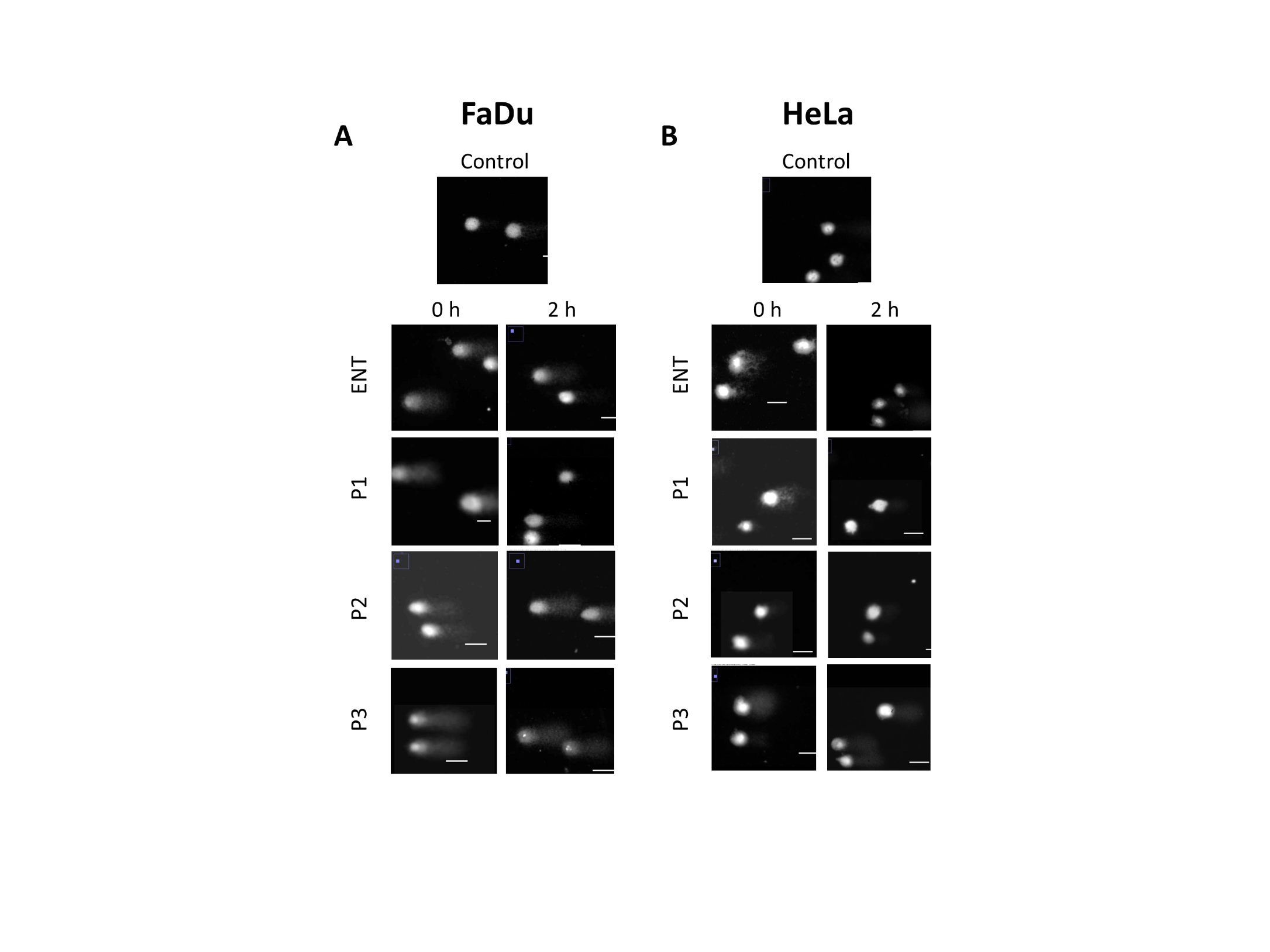
**

**Supplementary Figure 7. Protons with increasing LET lead to generation of persistent ALS/SSB-containing DNA damage in FaDu and HeLa cells.** (A) FaDu or (B) HeLa cells were irradiated (4 Gy) with entrance dose 28 MeV protons or the different positions across a pristine Bragg peak with increasing LET. Cells were then incubated for the respective time points to enable DNA repair, and DNA damage was measured by the alkaline comet assay. Respective images of DNA from unirradiated cells (Control), and those immediately and 2 hours post-irradiation, are shown.

**
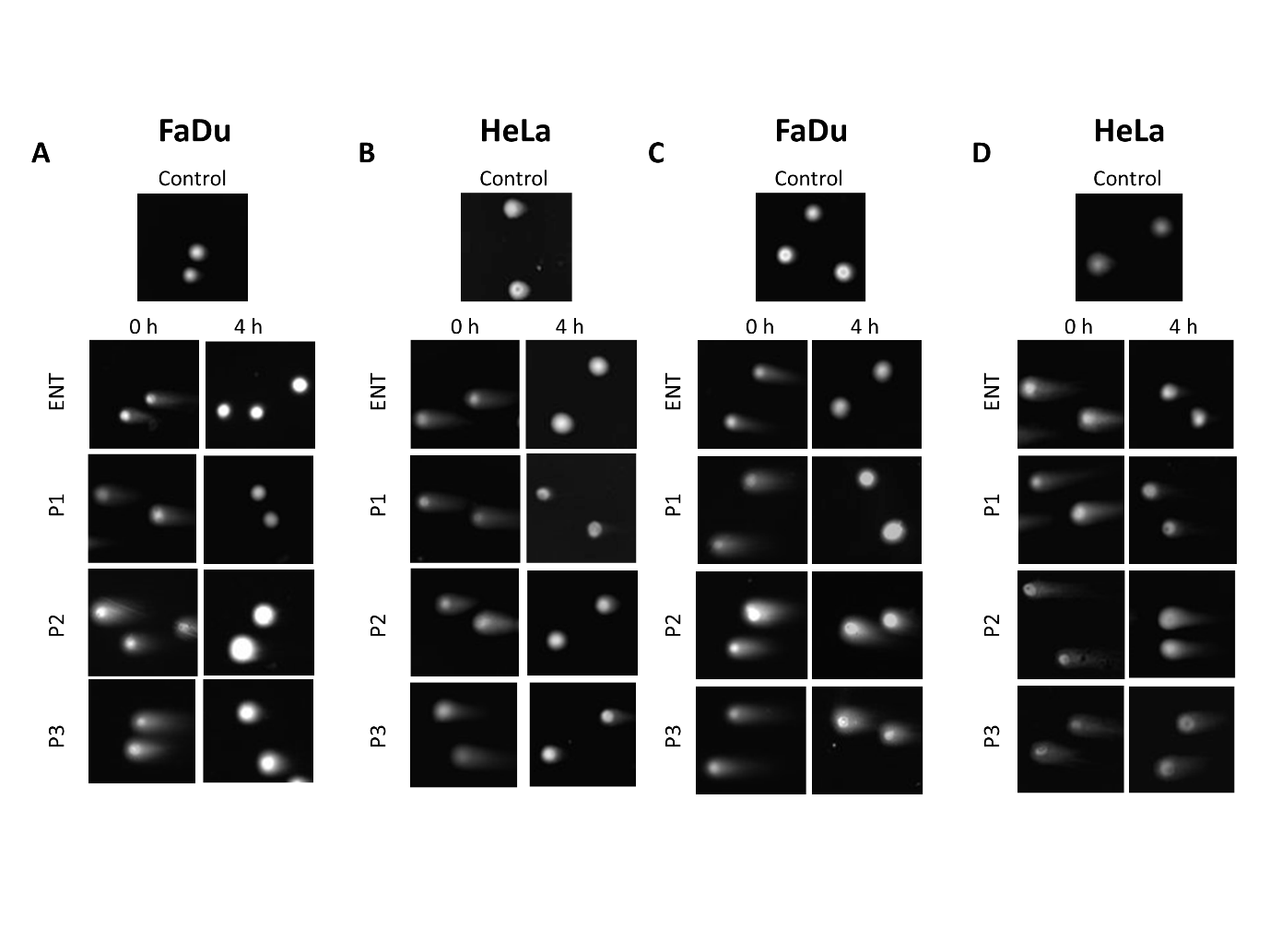
**

**Supplementary Figure 8. Protons with increasing LET lead to generation of persistent CDD in FaDu and HeLa cells.** (A and C) FaDu or (B and D) HeLa cells were irradiated (4 Gy) with entrance dose 28 MeV protons or the different positions across a pristine Bragg peak with increasing LET. Cells were then incubated for the respective time points to enable DNA repair, and DNA damage was measured at various time points post-irradiation by the enzyme modified neutral comet assay following incubation in the (A-B) absence (revealing DSBs) or (C-D) presence (revealing CDD; as indicated by mod) of the recombinant enzymes APE1, NTH1 and OGG1. Respective images of DNA from unirradiated cells (Control), and those immediately and 4 hours post-irradiation, are shown.

**Supplementary Table 1.** Analysis of chromosomal aberrations of HNSCC and HeLa cells treated with protons of increasing LET.

**
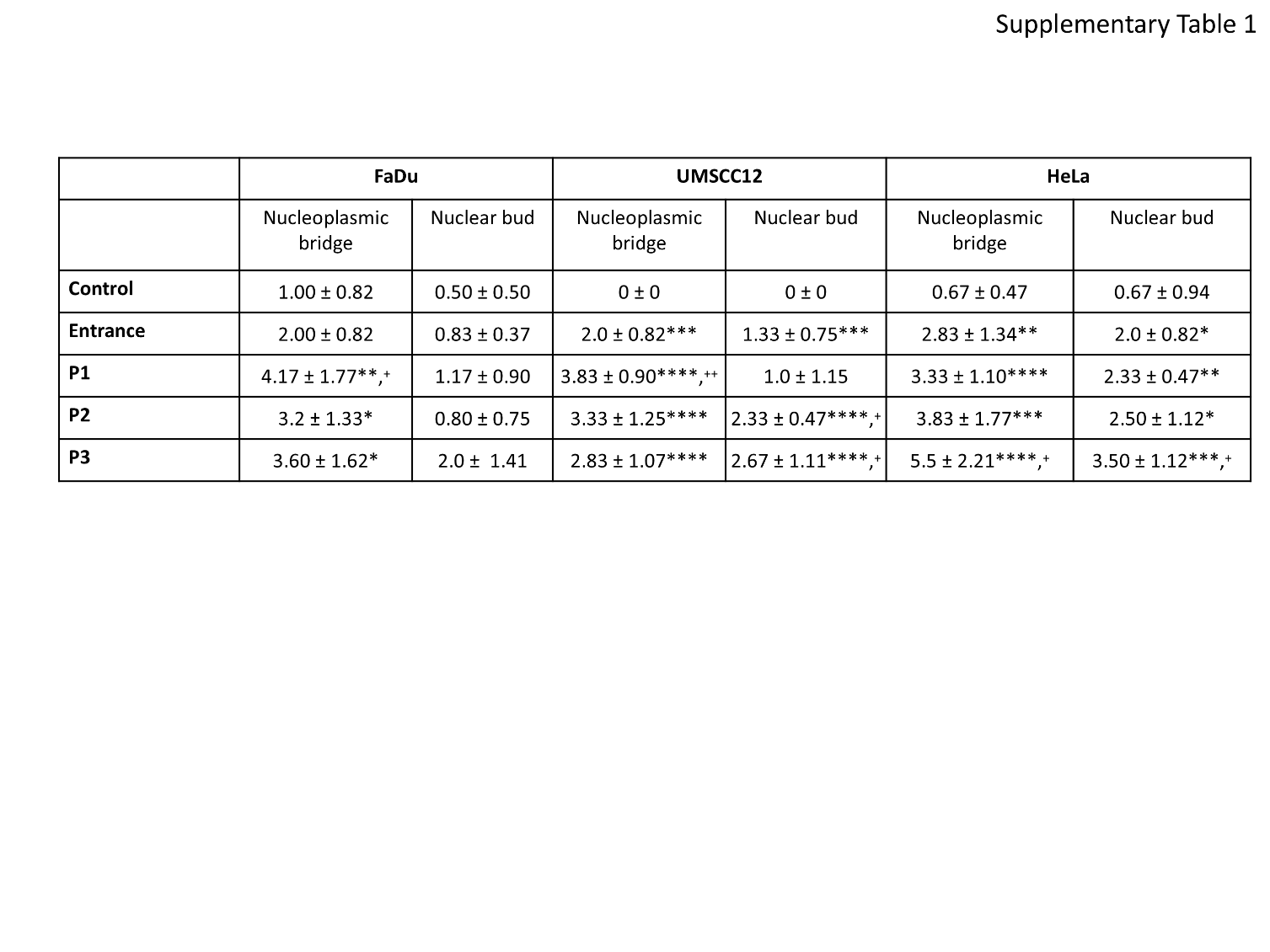
**

Statistical analysis was performed using a one sample *t*-test comparing aberrations from the above irradiated conditions versus the unirradiated control (*p<0.05, **p<0.01, ***p<0.005, ****p<0.001), or the positions relative to the Bragg peak versus entrance dose protons (^+^p,0.05, ^++^p<0.01).

**Supplementary Table 2.** Analysis of cell death of HNSCC and HeLa cells treated with protons of increasing LET. Shown are numbers of (A) apoptotic/necrotic cells and (B) mitotic figures.

**
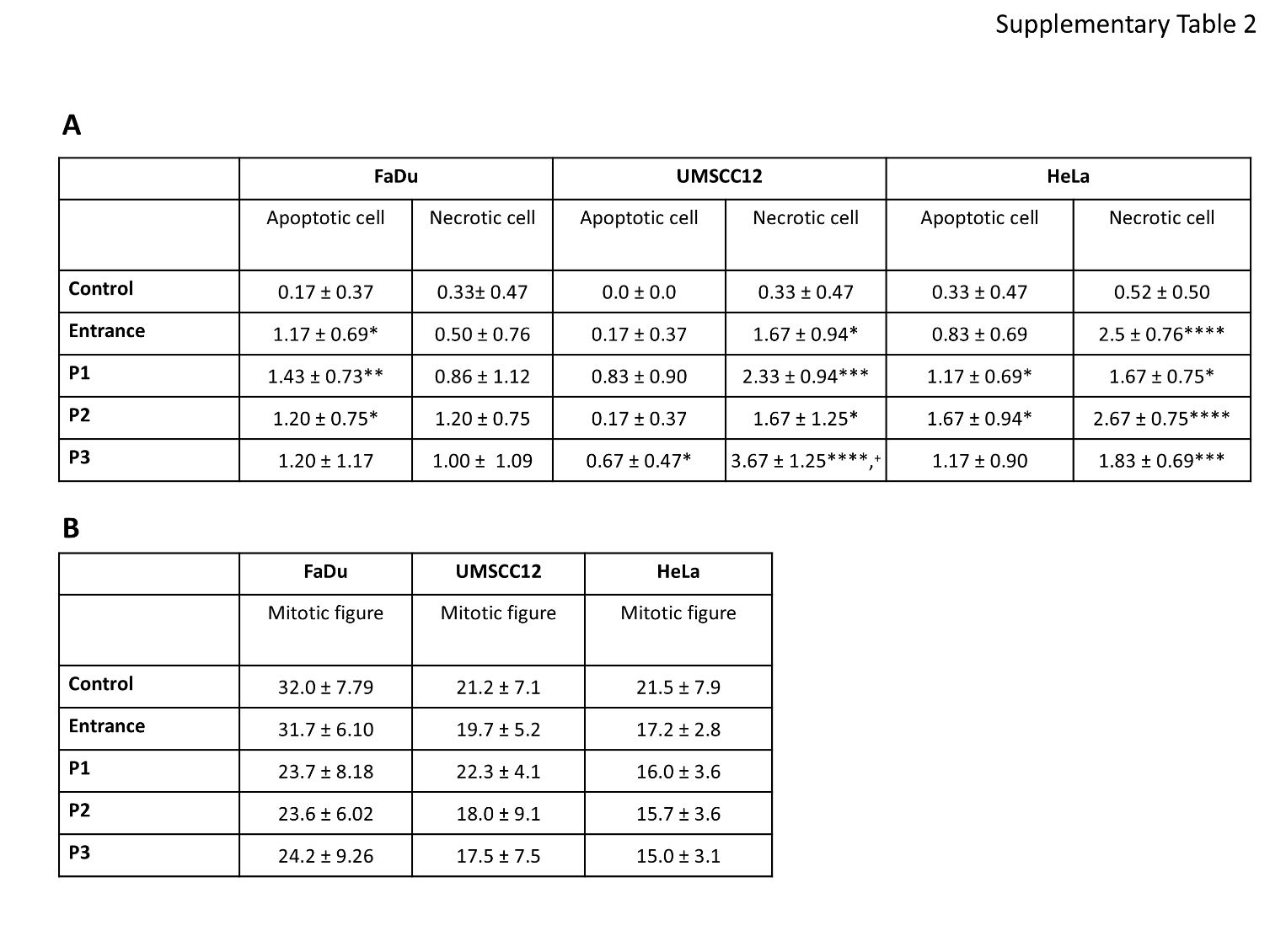
**

Statistical analysis was performed using a one sample *t*-test comparing aberrations from the above irradiated conditions versus the unirradiated control (*p<0.05, **p<0.01, ***p<0.005, ****p<0.001), or the positions relative to the Bragg peak versus entrance dose protons (^+^p,0.05, ^++^p<0.01).
